# Urinary *N*-acetyl-β-d-glucosaminidase, an early marker of diabetic kidney disease, might reflect glucose excursion in patients with type 2 diabetes

**DOI:** 10.1097/MD.0000000000004114

**Published:** 2016-07-08

**Authors:** So Ra Kim, Yong-ho Lee, Sang-Guk Lee, Eun Seok Kang, Bong-Soo Cha, Jeong-Ho Kim, Byung-Wan Lee

**Affiliations:** a Division of Endocrinology and Metabolism, Department of Internal Medicine, Graduate School, Yonsei University College of Medicine; b Severance Hospital; c Department of Laboratory Medicine, Yonsei University College of Medicine, Seoul, Republic of Korea.

**Keywords:** glycated albumin, *N*-acetyl-β-d-glucosaminidase, type 2 diabetes

## Abstract

Recently, several renal tubular damage markers have gained considerable attention because of their clinical implications as sensitive and specific biomarkers for early stage diabetic kidney disease. However, little is known about the demographic and glucometabolic factors affecting levels of urinary *N*-acetyl-β-d-glucosaminidase (NAG), a marker of proximal tubular damage, in type 2 diabetes mellitus (T2DM).

The aim of this study was to investigate the clinical relevance of urinary NAG with regard to demographic and glucometabolic parameters, as well as nephropathic parameters, by comparing the glomerulopathic marker of albuminuria.

In this retrospective cross-sectional study, we enrolled a total of 592 patients with either prediabetes (N = 29) or T2DM (N = 563). Glucometabolic parameters (glucose, hemoglobin A1c, glycated albumin [GA], insulin, C-peptide, homeostasis model assessment [HOMA] of insulin resistance, HOMA-β, postprandial C-peptide-to-glucose ratio [PCGR], and urinary glucose-to-creatinine ratio) and nephropathic parameters (urinary NAG, albumin-to-creatinine ratio [ACR], and estimated glomerular filtration rate) were measured.

The levels of urinary NAG showed moderate positive correlation with the levels of urinary ACR in T2DM (r = 0.46). In correlation analysis, urinary NAG was more strongly correlated with body mass index (BMI) (r = −0.22; *P* < 0.001 vs. r = −0.02; *P* = 0.74), plasma stimulated glucose (r = 0.25; *P* < 0.001 vs. r = 0.08; *P* = 0.10), GA (r = 0.20; *P* < 0.001 vs. r = 0.13; *P* = 0.01), PCGR (r = −0.17; *P* = 0.001 vs. r = −0.09; *P* = 0.11), and HOMA-β (r = −0.10; *P* = 0.05 vs. r = −0.02; *P* = 0.79) than urinary ACR. In multiple regression analysis, age, lower BMI, stimulated glucose, GA, and urinary ACR predicted increased urinary NAG.

In conclusion, increase in urinary NAG may be related to glycemic parameters reflecting glucose fluctuation and decreased insulin secretory capacity in patients with T2DM. Further longitudinal, prospective studies are needed to investigate a causal relationship between glucose fluctuations, renal tubular damage, and other vascular complications of diabetes.

## 1 Introduction

Diabetic kidney disease (DKD) occurs in 20% to 40% of patients with diabetes and is the leading cause of chronic kidney disease (CKD) as well as end-stage renal disease.^[1]^ Although the urinary albumin excretion rate is commonly used as the earliest clinical index of CKD,^[2]^ it faces some challenges as an indicator for CKD. Some patients undergo decreases in glomerular filtration rate without increases in urinary albumin excretion rate.^[3]^ The nephron, which is composed of the renal corpuscle and renal tubule, is the basic structural and functional unit of the kidney. Along with the importance of glomerular damage in DKD, renal tubulointerstitial injuries may also play an important role in the development and progression of DKD.^[4,5]^ Recently, several tubular damage markers, including kidney injury molecule-1, neutrophil gelatinase-associated lipocalin, *N*-acetyl-β-d-glucosaminidase (NAG), heart fatty acid–binding protein, and cystatin C, have gained considerable attention because of their clinical implications as sensitive and specific biomarkers for predicting the development and progression of early stage DKD.^[6–10]^

The urinary enzyme NAG is found in the lysosomes of proximal tubule epithelial cells. Because of its high molecular weight of 130 kDa, plasma NAG cannot be filtered through the glomerulus and its increase in urine is caused exclusively by its secretion from proximal tubular cell lysosomes by proximal tubular cell injury.^[11]^ Previous studies^[6,12]^ reported that compared to controls, increases in urinary NAG excretion already occur in patients with normal to mildly increased albuminuria with type 2 diabetes mellitus (T2DM). Based on these findings, we could assume that subclinical tubular dysfunction might develop earlier than glomerular damage. Several studies investigated the relation of urinary NAG excretion to the severity of kidney disease as assessed by albuminuria and the estimated glomerular filtration rate (eGFR) in patients with diabetes.^[6,9]^ However, little research has been done regarding the relation between urinary NAG excretion and glycemic and insulin-related parameters. Studies have shown only that urinary NAG excretion gradually increases with increases in duration of diabetes,^[13]^ fasting plasma glucose levels,^[14]^ or hemoglobin A_1c_ (HbA_1c_) levels.^[15]^

The aim of this study was to investigate the clinical relevance of urinary NAG, a renal tubulopathic marker, regarding demographic and glucometabolic parameters, as well as nephropathic parameters, by comparing the glomerulopathic marker of albuminuria.

## 2 Methods

### 2.1 Study participants

In a retrospective cross-sectional design, we recruited participants with prediabetes or T2DM who attended the Severance Hospital Diabetes Center between March 2015 and December 2015, and had been tested for serum glycated albumin (GA), HbA_1c_, urinary NAG, and a standardized liquid meal test. Participants who met the following criteria were excluded: younger than 20 years of age, having type 1 diabetes, taking sodium–glucose cotransporter 2 inhibitor, and pregnant women. Participants were assigned to either a prediabetes or a T2DM group. Prediabetes was defined as HbA_1c_ of 5.7% to 6.4% (38.8–46.4 mmol/mol). T2DM was defined on the basis of the participant's use of insulin or oral hypoglycemic agents or HbA_1c_ ≥6.5% (47.5 mmol/mol). Age, sex, body mass index (BMI), smoking habits, blood pressure, duration of diabetes, and antidiabetic drugs were recorded. The Institutional Review Board at Severance Hospital approved this study protocol (4-2015-0828). Written informed consent for this study was not required by the Institutional Review Board because the database was accessed only for analysis purposes and personal information was not used.

### 2.2 Measurements of blood glucometabolic parameters

Following an overnight fast, blood samples were collected from participants before (0 minute; designated as basal) and after (90 minutes; designated as stimulated) ingestion of 2 cans (total 400 mL, 400 kcal, 18 g fat, 44 g carbohydrate, and 20 g protein) of a standardized mixed meal (Mediwell Diabetic Meal, Meail Dairies Co, Yeongdong-gun, Chungbuk, Republic of Korea) to measure glucose and insulin/C-peptide, and to perform other chemistry tests. Pancreatic β-cell function and insulin sensitivity were assessed using the following indices^[16]^: homeostasis model assessment (HOMA)-β = ([basal insulin (pmol/L) × 3.33]/[basal glucose (mmol/L) − 3.5]), homeostasis model assessment of insulin resistance = ([basal insulin (pmol/L) × basal glucose (mmol/L)]/135), C-peptide increment (ΔC-peptide = [stimulated C-peptide (nmol/L) − basal C-peptide (nmol/L)]), and insulin increment (Δinsulin = [stimulated insulin (pmol/L) − basal insulin (pmol/L)]). Postprandial C-peptide-to-glucose ratio (PCGR) was defined as follows^[17]^: (stimulated C-peptide [ng/mL]/stimulated glucose [mg/dL]) × 100. The eGFR was derived from the Chronic Kidney Disease Epidemiology Collaboration (CKD-EPI) equation and also from the Modification of Diet in Renal Disease (MDRD) equation for the Korean population.^[18,19]^ HbA_1c_ was measured by immunoassay using an Integra 800 CTS (Roche, Hercules, CA). Serum GA levels were determined by an enzymatic method (LUCICA GA-L, Asahi Kasei Pharma Co, Tokyo, Japan), using a Hitachi 7600 autoanalyzer (Hitachi Ltd, Tokyo, Japan). Serum glucose and creatinine were also measured using the Hitachi 7600 analyzer (Hitachi Ltd). For serum creatinine, a compensated kinetic Jaffe method (Clinimate CRE, Sekisui Medical Co Ltd, Tokyo, Japan) was used, in which the creatinine concentration has been standardized to isotope dilution mass spectrometry. Serum insulin and C-peptide were measured by an electrochemiluminescence immunoassay with a cobas e601 analyzer (Roche Diagnostics, Basel, Switzerland). Cystatin C was measured by an immunoturbidimetric method using a cobas c501 analyzer (Roche Diagnostics).

### 2.3 Measurements of urinary glomerular and tubular damage markers

Urinary NAG, albumin, glucose, and creatinine levels were measured in the fasting morning spot urine sample that was obtained from each participant. Urinary NAG, albumin, and glucose levels were expressed as urinary NAG-to-creatinine ratio, albumin-to-creatinine ratio (ACR), and glucose-to-creatinine ratio (GCR) to minimize the influence of the variations of kidney function. Urinary NAG activity was considered abnormal when >4 U/g creatinine.^[20]^ Categories of albuminuria were defined as follows^[21]^: normal to mildly increased albuminuria, ACR <3.0 mg/mmol; moderate albuminuria, 3.0 ≤ ACR ≤ 30 mg/mmol; and severe albuminuria, ACR >30 mg/mmol. Urine level of NAG was measured using a reagent from Nittobo Medial Co, Ltd (Tokyo, Japan), and a JCA-BM 6010/c automated chemistry analyzer (JEOL Ltd, Tokyo, Japan). Urine level of albumin was measured by an immunoturbidimetric method using AU680 automated chemistry analyzer (Beckman Coulter, Inc, Brea, CA). Urine level of creatinine was also measured using the AU680 analyzer (Beckman Coulter, Inc) by kinetic Jaffe method.

### 2.4 Statistical analyses

All statistical analyses were performed using SPSS version 20.0 for Windows (IBM Corp, Armonk, NY). The normality test was performed for all continuous variables. The data are presented as mean ± standard deviation for normally distributed continuous variables and median (interquartile range) for non-normally distributed continuous variables. Categorical data are expressed as numbers and percentages. The characteristics of the study participants were analyzed according to their diabetes status or urinary NAG levels using the Mann–Whitney *U* or 2-sample Student *t* test for continuous variables and the Pearson χ^2^ test for categorical variables. Correlations between urinary NAG or ACR and other parameters were analyzed with the Spearman or Pearson correlation coefficient. Stepwise multiple linear regression analysis was performed on logarithm-transformed values of urinary NAG in order to model the relationship between the urinary NAG and demographic, glycemic, insulin secretory/resistant, and nephropathic parameters. Distributions of urinary NAG concentrations in T2DM according to tertiles of GA, stimulated glucose, and PCGR were examined using box-and-whisker plots. Outliers of urinary NAG >100 U/g Cr (N = 3) were excluded in the graph. Significance was tested using the Mann–Whitney *U* test. All *P* values <0.05 were considered statistically significant.

## 3 Results

### 3.1 Characteristics of the study participants

A total of 592 participants (370 men and 222 women) were enrolled in this study. The demographic and laboratory characteristics of the participants according to glucose tolerance (29 with prediabetes, 563 with T2DM) are shown in Table 1. The age, sex distribution, BMI, smoking status, and systolic blood pressure were similar between groups. The median age of participants with T2DM was slightly higher than that of participants with prediabetes, but did not reach significance. The participants with T2DM had significantly higher levels of blood and urinary glucose (basal and stimulated glucose, GA, HbA_1c_, and urinary GCR) than those with prediabetes. In the group with T2DM, the median duration of diabetes and HbA_1c_ were 8.75 years and 7.30% (56.3 mmol/mol), respectively.

**Table 1:**
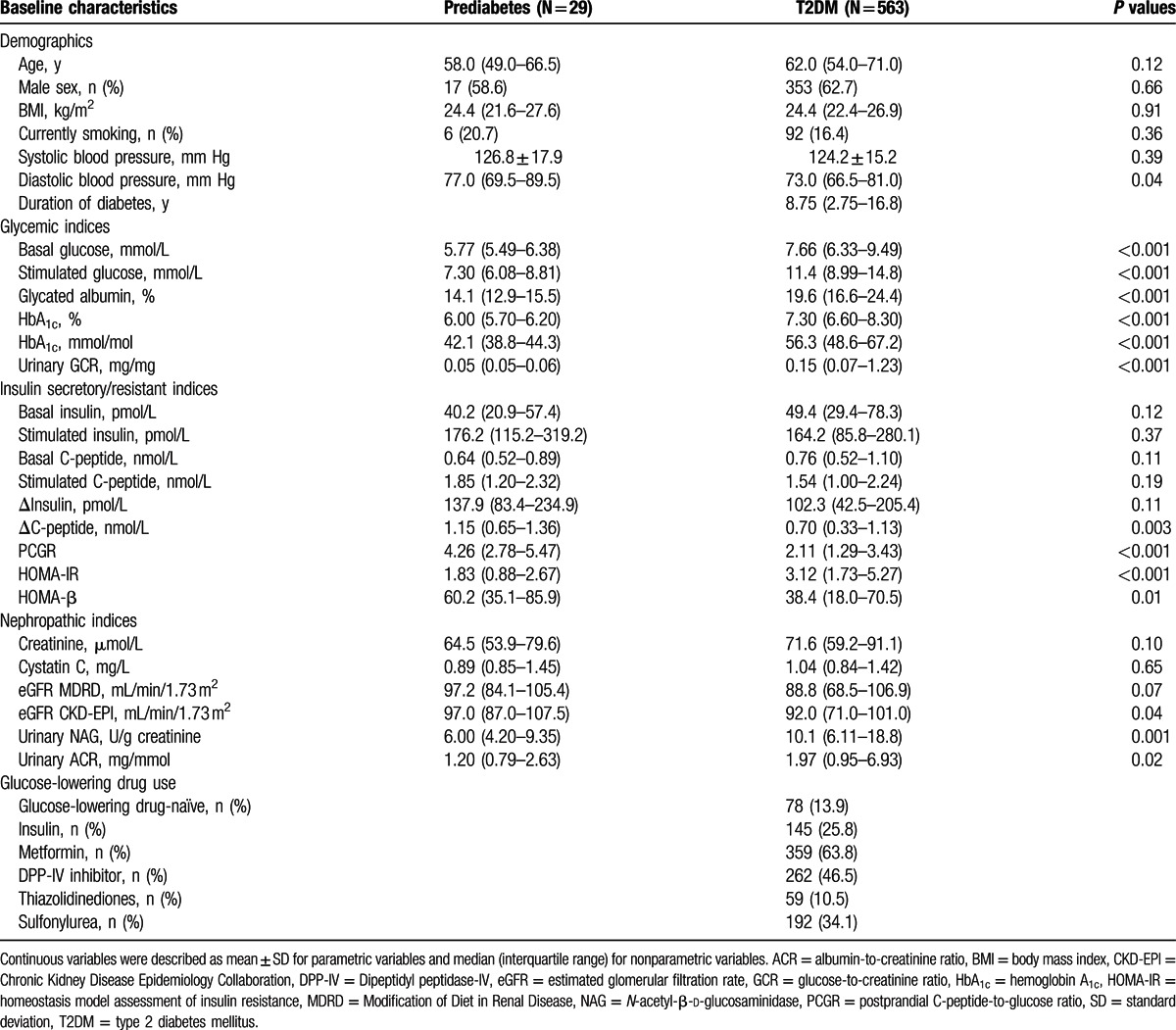
Baseline demographic and laboratory characteristics of participants.

With respect to insulin–glucose homeostasis, the levels of basal and stimulated insulin and C-peptide did not significantly differ between the groups, but insulin secretory parameters, including ΔC-peptide, PCGR, and HOMA-β, were significantly lower in participants with T2DM compared to those in participants with prediabetes. In addition, the insulin-resistant parameter of HOMA of insulin resistance in participants with T2DM was significantly higher than in participants with prediabetes.

Regarding renal function and nephropathic indices, the levels of serum creatinine, serum cystatin C, and eGFR MDRD did not differ between the groups, but the eGFR values calculated by CKD-EPI equation were significantly lower in the T2DM than in the group of prediabetes. The urinary ACR and NAG were significantly increased in participants with T2DM compared with in those with prediabetes.

### 3.2 Correlation between urinary NAG or ACR and other parameters

Of the various metabolic and nephropathic parameters in participants with T2DM, urinary NAG showed a moderate positive relationship with urinary ACR (r = 0.458, *P* < 0.001) in the Spearman correlation analyses. We therefore evaluated the correlation between the aforementioned parameters and both urinary NAG and ACR, which is regarded as a marker of glomerular damage in diabetic nephropathy, to compare the correlation coefficients with those of urinary NAG (Table 2). Both urinary NAG and ACR were positively correlated with age, duration of diabetes, HbA_1c_, urinary GCR, and serum cystatin C. In contrast to urinary ACR, urinary NAG showed significant correlations with BMI, basal glucose, stimulated glucose, GA, PCGR, and HOMA-β. In addition, urinary ACR showed significant correlations with systolic blood pressure, serum creatinine, and eGFR MDRD, whereas urinary NAG did not show these correlations. Urinary NAG was not significantly correlated with eGFR MDRD (r = −0.069, *P* = 0.10), but was significantly correlated with eGFR CKD-EPI (r = −0.168, *P* < 0.001).

**Table 2:**
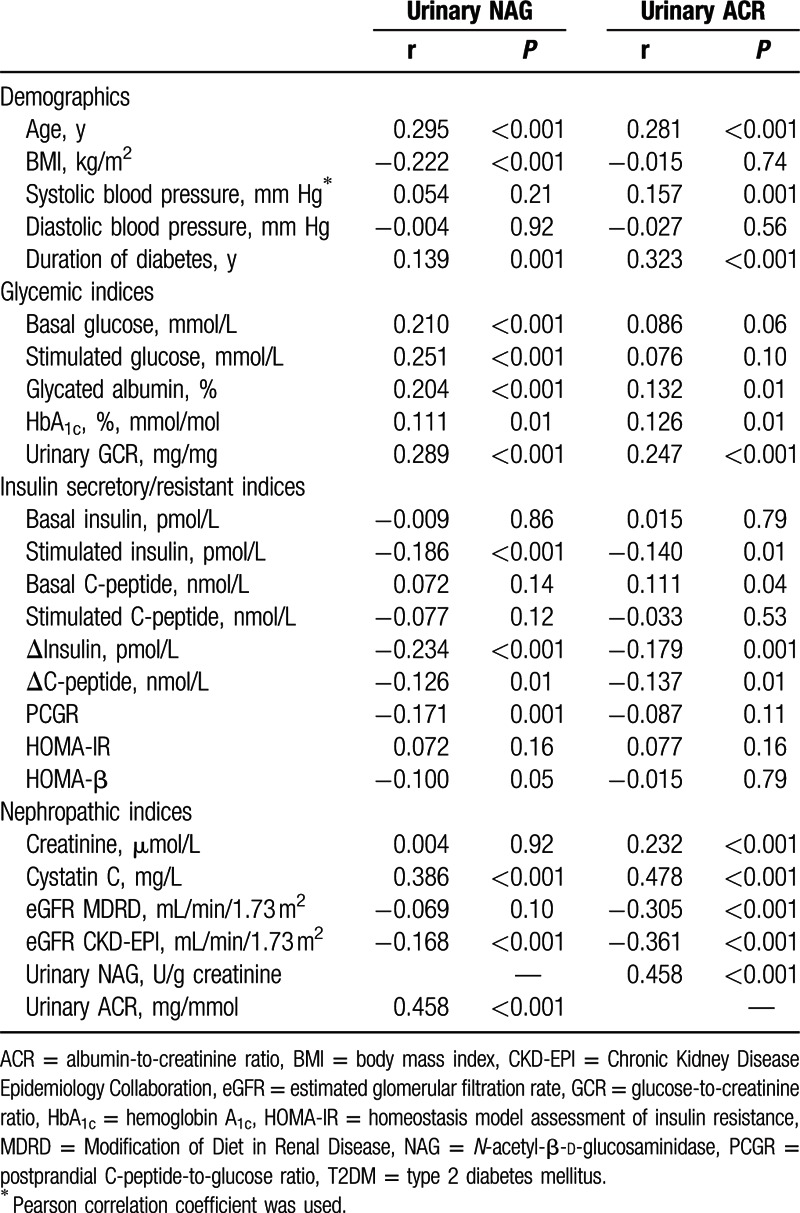
Correlation between urinary NAG or ACR and other parameters in T2DM (N = 563).

### 3.3 Characteristics of participants with increased urinary NAG levels in normal to mildly increased albuminuric T2DM

To exclude the effects of albuminuria, we classified the participants with normoalbuminuria into 2 groups according to urinary NAG levels (normal vs. increased [>4 U/g creatinine]) (Table 3). The median values of urinary NAG in participants with normal and increased urinary NAG levels were 3.33 (2.73–3.77) and 8.84 (6.31–14.7) U/g creatinine, respectively. The participants with increased urinary NAG levels were older and had significantly higher levels of basal glucose, stimulated glucose, GA, and urinary GCR than those with normal urinary NAG levels. Insulin secretory/resistant indices and values of eGFR did not differ between the 2 groups.

**Table 3:**
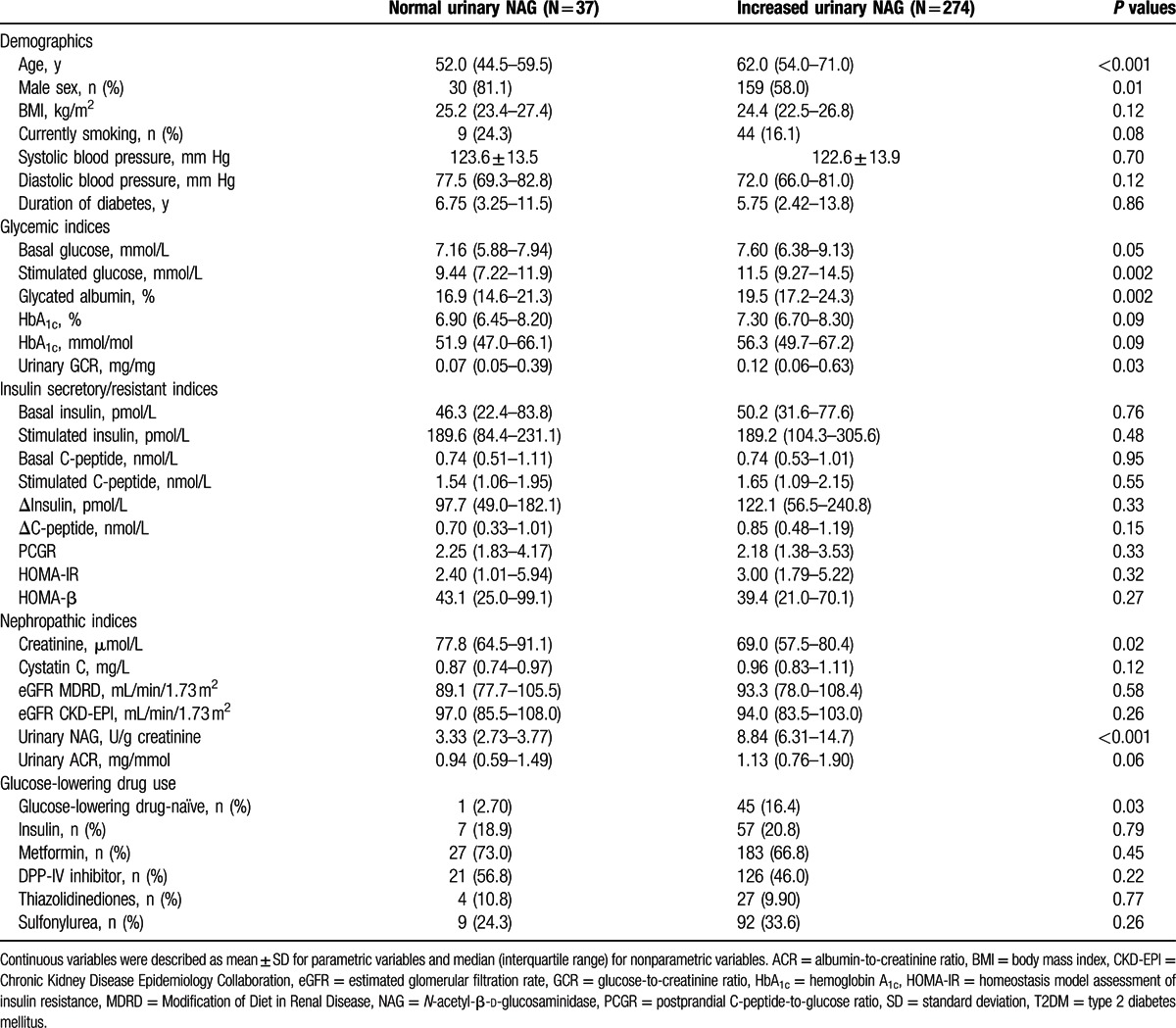
Characteristics of participants with respect to normal or increased urinary NAG levels in normal to mildly increased albuminuric T2DM (N = 311).

### 3.4 Determinants of urinary NAG in participants with T2DM by multiple linear regression analysis

To determine the factors that were predictive of renal tubulopathy in participants with T2DM, we performed multiple linear regression analyses (Table 4). The analyses were performed separately for 4 models. In model 1, we entered demographic parameters, including age, sex, BMI, smoking status, and duration of diabetes, as independent factors and urinary NAG as a dependent factor. Age (Standardized Beta [STD β] = 0.25, *P* < 0.001] and BMI (STD β = −0.11, *P* = 0.01) were significantly associated with urinary NAG. In model 2, with inclusion of age, sex, and glycemic- and insulin-related parameters, such as basal glucose, stimulated glucose, Δinsulin, ΔC-peptide, PCGR, HOMA-β, urinary GCR, sulfonylurea use, and insulin use, 2 parameters of age (STD β = 0.27, *P* < 0.001) and stimulated glucose (STD β = 0.24, *P* < 0.001) were significantly associated with urinary NAG. In model 3, regarding glomerulotubular damage-related parameters, such as age, sex, eGFR CKD-EPI, urinary ACR, serum cystatin C, GA, HbA_1c_, sulfonylurea use, and insulin use, 3 parameters of age (STD β = 0.30, *P* < 0.001), GA (STD β = 0.25, *P* < 0.001), and urinary ACR (STD β = 0.36, *P* < 0.001) were significantly associated with urinary NAG. Finally, in model 4, we entered the significant variables in models 1 to 3 as independent factors. Age (STD β = 0.27, *P* < 0.001), lower BMI (STD β = −0.09, *P* = 0.04), stimulated glucose (STD β = 0.13, *P* = 0.01), GA (STD β = 0.12, *P* = 0.02), and urinary ACR (STD β = 0.26, *P* < 0.001) predicted increased urinary NAG.

**Table 4:**
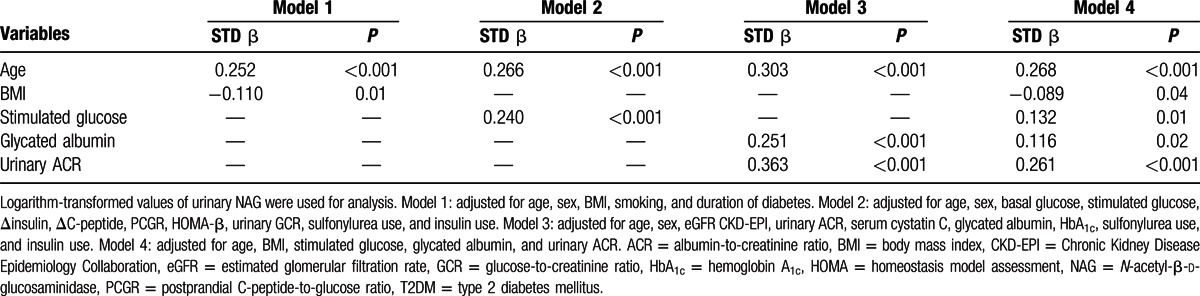
Multiple linear regression models for urinary NAG in T2DM.

**Figure 1:**
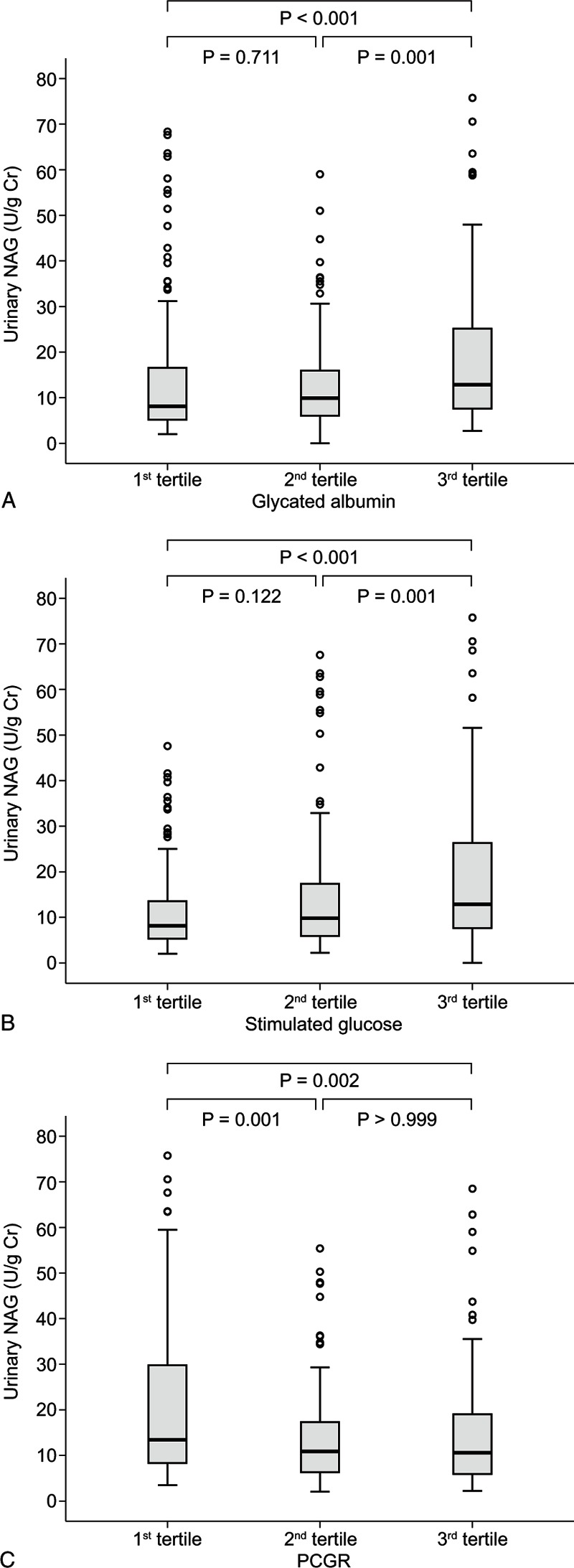
Boxplots of urinary NAG concentrations in T2DM according to tertiles of glycated albumin (A), stimulated glucose (B), and PCGR (C). Circles represent outliers. NAG = *N*-acetyl-β-d-glucosaminidase, PCGR = postprandial C-peptide-to-glucose ratio, T2DM = type 2 diabetes mellitus.

Based on the Spearman correlation and multiple linear regression analyses, the participants with T2DM were divided into 3 groups according to tertile of levels of GA, stimulated glucose, and PCGR. The levels of urinary NAG tended to increase with increase of GA or stimulated glucose, and decrease with increases of PCGR (Fig. 1).

## 4 Discussion

Evidence has accumulated on the clinical relevance of NAG as tubular damage marker.^[22–24]^ However, optimal interpretation of urinary NAG as renal tubulopathic index in the context of glucose metabolism has not been fully investigated. Based on previous reports that increased urinary NAG excretion could be found in patients with normal to mildly increased albuminuric T2DM,^[6,9]^ we hypothesized that subclinical tubular dysfunction assessed by urinary NAG might be more sensitive than glomerular deterioration assessed by either eGFR or albuminuria in the context of glucose metabolism. To examine this hypothesis, we investigated clinical relevance of urinary NAG by analyzing various parameters of glucose metabolism and nephropathies. In this study, we demonstrated 4 main findings. First, the levels of urinary NAG showed moderate positive correlation with the levels of urinary ACR. Second, the increase in urinary NAG might better reflect the decrease in eGFR calculated by the CKD-EPI equation. Third, increases in urinary NAG were associated with GA and stimulated or postprandial glucose in patients with T2DM. Fourth, urinary NAG levels were positively correlated with age and duration of diabetes and negatively correlated with BMI.

With respect to the levels of urinary NAG in conjunction with the stages of albuminuria, it is well known that urinary levels of NAG are higher when patients have more aggravated albuminuria.^[6,9,13,15]^ In contrast to the conventional linear relationship between urinary NAG and albuminuria, urinary NAG could be significantly increased even in patients with normal to mildly increased albuminuric T2DM compared with in patients without diabetes.^[6,9]^ Tanaka et al demonstrated that 62% of patients with normal to mildly increased albuminuric T2DM showed a significant increase in urinary NAG.^[25]^ Similar to previous work, our study results showed that urinary NAG showed a moderate positive correlation (r = 0.458) with urinary ACR and 88% of 311 participants with normal to mildly increased albuminuria with T2DM had increased urinary NAG excretion. Although there are results that either the tubulotoxic effect of urinary albumin^[26]^ or the exceeding tubular reabsorption capacity of filtered albuminuria^[11]^ might account for significant correlation between increased levels of urinary NAG and albuminuria in moderate to severe albuminuric stages, they could not explain the finding of high levels of NAG in the normoalbuminuric stage. A possible explanation for these findings might be inferred from a previous histological study^[27]^ in which the proximal tubular basement membrane was shown to be already thickened in patients with normoalbuminuria with diabetes compared with healthy control participants. This study has suggested that tubular damage occurs early in the course of DKD and is not merely secondary to glomerular damage. Based on these findings, we suggest that urinary NAG might be a sensitive urinary biomarker for early detection of DKD.

With respect to the correlation between urinary NAG and eGFR, it is not clear whether an increase in urinary NAG predicts reduction in eGFR.^[28]^ Nauta et al^[9]^ showed the lost association of eGFR with the levels of urinary NAG after adjusting for age, sex, and albuminuria. Furthermore, Fu et al^[6]^ demonstrated that levels of urinary NAG were inversely related with eGFR only in patients with macroalbuminuria. Because NAG cannot be filtered through the glomerulus, it might be more logical that the presence of urinary NAG is exclusively caused by its secretion from proximal tubular cells, and an increase in urinary NAG along with a decrease in eGFR implies glomerulotubular damage. However, in this study, we found that the urinary NAG was slightly negatively correlated with eGFR calculated by CKD-EPI equation but not by MDRD equation. The plausible explanation for this finding might be dependent on which kind of the equation formula for calculating eGFR was used. Previous studies calculated eGFRs based on MDRD equation and Macisaac formula, respectively. Regarding eGFR calculation equation, we previously demonstrated and suggested that the CKD-EPI equation might more accurately stratify earlier stage CKD among patients with T2DM than the MDRD equation.^[19]^ Based on these findings, we suggest that the nephropathic biomarker of urinary NAG and eGFR calculated by the CKD-EPI equation might be more sensitive for early detection of DKD in patients with T2DM.

With respect to the influences of glucometabolic parameters on urinary NAG, it was reported that increased urinary NAG excretion was present in patients with poorly controlled glycemia.^[29,30]^ Furthermore, a decrease in urinary NAG excretion could be achievable in patients with T2DM after intervention of short-term intensified glycemic control.^[14]^ In this study, we demonstrated that stimulated glucose, GA, and decreased insulin secretory function predicted the diabetic renal tubulopathy assessed by urinary NAG. To our knowledge, this is the first study on the correlation between urinary NAG with GA and insulin secretory indices. Regarding plausible explanations for associations between high glucose in plasma or urine and increases in urinary NAG excretion, both physiologic increases in urinary NAG for metabolizing urinary glucose and the nephrotoxic effect of glycated end products on renal proximal tubules could be possible. NAG is an enzyme involved in carbohydrate metabolism. When the proximal tubules are exposed to high urinary glucose, NAG might be secreted more in the urine, depending on urinary glucose concentrations.^[31]^ In addition, the peptides derived from advanced glycation end products might have a potential nephrotoxic effect on the proximal tubule, thus contributing to the occurrence of proximal tubule injury^[32]^ or reflecting diabetic nephropathy. We previously suggested^[33]^ that serum GA was more associated with postprandial glucose than with fasting glucose, and might be a useful index for monitoring glycemic control in patients with T2DM who have fluctuating and poorly controlled glycemic excursions. The clinical relevance of GA might be attributed to the pathophysiologic phenomenon that increased GA over HbA_1c_ was significantly correlated with insulin secretory β-cell function assessed by PCGR and HOMA-β and increased as duration of diabetes increased.^[34]^ In our study, we also demonstrated that urinary NAG was more significantly correlated with serum GA than with urinary ACR. Previous reports showed that GA, which is influenced by insulin secretory dysfunction, is not only a useful glycemic index for fluctuating and poorly controlled diabetes but also an atherogenic protein in development of diabetic vascular complications.^[34–36]^ Accumulating evidence also suggests that urinary NAG is correlated or associated with vascular complications of T2DM, not only nephropathy but also retinopathy,^[37]^ neuropathy,^[38]^ and macrovascular disease.^[39]^ We propose that glucose excursion might be an explanatory factor for associations between urinary NAG and vascular complications in type 2 diabetes.

With respect to the correlation between urinary NAG and diabetes-related variables, our data showed that urinary NAG was associated with age, duration of diabetes, and, interestingly, BMI. Because diabetes complications are closely associated with old age and diabetes duration, the positive correlation between urinary NAG and both age and diabetes duration is acceptable without hesitation. However, the negative correlation between urinary NAG and BMI might result from the fact that Korean patients with diabetes have relatively low BMI with insulin secretory dysfunction compared to those in Western countries.^[40]^ Further studies are needed to investigate how these factors contribute to tubular damage.

The current study had several strengths. First, we conducted the study with a relatively large number of patients with dysregulated glucose control, which strengthens the statistical reliability of the results. Second, we investigated the clinical relevance of urinary NAG, a renal tubulopathic marker, regarding demographic, glucometabolic, and nephropathic parameters by comparing glomerulopathic marker of albuminuria, which might reinforce comparability. Additionally, we performed a standardization of glucohomeostasis markers, including insulin secretory and sensitivity, using a standardized mixed meal tolerance test. However, the current study also has some limitations. First, because of the inherent drawback of cross-sectional study, we could not validate if there is a causal relationship between levels of GA and either initiation or progression of diabetic tubulopathy. Second, the values of Spearman correlation coefficient between urinary NAG and clinicobiochemical parameters, including age, BMI, GA, and PCGR, were statistically significant but slight to fair.

In conclusion, we evaluated clinical relevance of urinary NAG on demographic, glucometabolic, and nephropathic parameters, and revealed a novel finding that urinary NAG was associated with postprandial glucose and GA in patients with T2DM. Furthermore, urinary NAG might be a more sensitive urinary biomarker than urinary albumin for early detection of DKD. From the results of the current study and based on previous reports on GA, we postulate that an increase in urinary NAG may be related to plasma glucose fluctuation and decreased insulin secretory capacity in patients with T2DM. Further longitudinal prospective studies are needed to investigate a causal relationship between glucose fluctuations, renal tubular damage, and other vascular complications of diabetes.
